# Improving Urine Sample Efficacy as a Convenient Alternative for Invasive Samples in Molecular Diagnosis of Toxoplasmosis

**Published:** 2013

**Authors:** AA Eskandarian

**Affiliations:** Department of Parasitology & Mycology, Faculty of Medicine, Esfahan University of Medical Sciences, Esfahan, Iran

**Keywords:** Urine, Molecular diagnosis, Toxoplasmosis, Nested PCR

## Abstract

**Background:**

Diagnosis of some diseases is difficult due to invasive sampling. Urine has been candidate as a non-invasive and convenient alternative. It has many advantages and easy accessibility but some technical ills should be removed. Finding a suitable extraction method for improving urine DNA quantity and quality in altering invasive specimens for molecular diagnosis of some infectious diseases, was the main object of present research.

**Method:**

Toxoplasmosis was selected as an experimental model, regarding the congenital and ocular forms, its abundance and requirement to invasive sample for diagnosis. Samples prepared by adding some defined *Toxoplasma gondii* (RH strain) tachyzoites to normal urine. Several urine DNA extraction and purification methods comparatively were tested for finding the best one. The amount of extracted DNA assessed using Nanodrope spectrophotometer and a multiplex semi-nested PCR were designed for evaluating the results.

**Results:**

Urine samples with known number of tachyzoites were purified comparatively by five better methods. The results reviled that Cinnagen kit performed with more efficacies. It works well up to 1-5tachyzoites µl^−1^ of urine. The amount and quality of extracted DNA of more than 100 urine samples with defined tachyzoites were analyzed using a nested PCR method. Finally methods were enough sensitive to detect one tachyzoite DNA in final PCR reaction.

**Conclusion:**

This method was enough eligible and sensitive to perform molecular tests for different purposes of instance detecting toxoplasmosis by urine sample as a convenience and non invasive method; although it is better to perform some more experiments using patients samples comparing gold methods.

## Introduction

Urine is a biological waste liquid produced by kidneys subsequent of blood infiltration. The simplicity and convenience of urine for sampling, is a considerable priority of urine compare to invasive samples (1). The presence of short DNA fragments in human urine was reported by a cell biologist- David Tomei- and his colleagues in about two decades ago for first time (2). The urine could be used for different purposes such as diagnosis of congenital diseases before birth; organ implantation surveillance; cancer diagnosis; typing of microorganisms; drug resistance assessment; drug metabolism researches and so on.

The parasites are the infective agents that invade nearly all body tissues. Invasion of some parasites to central nerves system, optic system and fetuses, render them sometimes risky and life threatening. Their laboratory diagnosis yet depends on invasive specimen of infected tissues. The obtaining of those samples associated with hazards and side effects for patients. Due to invasive sampling methods, the investigators prefer to use urine sample that is safe and easy accessible (4–6). Nowadays urine sample used for diagnosis of several parasitic infections includes malaria, toxoplasmosis, amoebiasis, trichomoniasis, leishmaniasis, trypanosomiasis etc (3–10).

Botezatu and colleagues confirmed excretion of DNA related to injected bacteriophage and radiated Raji cells in urine of mice, using PCR method. They showed that kidneys purify blood DNAs to urine(2).

Finding of the relationship between a specific DNA in blood and urine needs to more experiments. Some of serum DNA pass the kidneys and inter to urine. Su and coworkers assessed the librated DNA (free DNA) in urine of five cases. The amount of DNA was about 40 – 200 ng ml^−1^ by specterophotometry. After electrophoresis on 8% polyacrylamid gel it revealed two different bands; 1- a heterogenic high band about 1000 bp and 2- a homogenic low band about 150 –250 bp (4, 11).

Molecular base techniques need to be enough and suitable amount of DNA. It should consider the time that urine sample could stored in cold circumstances without a serious damage to its DNA content (4–5, 8, 10–12).

According to Milde the extractable of urine DNA is between 53-200 ±4.55 ng ml^−1^ for women and 3- 50 ± 17.58 ng ml^−1^ for men (13).

This is a descriptive original study aimed to find some experiences toward using the urine sample as a good alternative for invasive samples.

Concentrations on *Toxoplasma gondii* as typical infectious agent lead to obtain some facts for using the urine as biological samples in some molecular based diagnostic methods.

## Materials and Methods

### Sample preparation

The normal human urine from midstream of some normal healthy people in sterile Falcon tube was used. As this study aimed to use the urine in different conditions, it used the *T. gondii* as a model and spiked it to normal urine in defined concentration and performed a great deal of tests.

### Parasite

The RH strain of *T. gondii* obtained from Pasteur Institute of Iran (Tehran) and maintained on small white laboratory mice with weekly passage.

### DNA Extraction

Several DNA extraction experiences by different methods were done for finding the best method and introducing different extraction methods for different conditions. These included:

#### 1- Funtes method 1996 (10)

This method designed for purification of *T. gondii* DNA from urine used directly for nested PCR. The author carried out this extraction method on toxoplasmosis patients’ urine.

#### 2- Holman method 2003 (14)

Holman used it for extraction of DNA of BK polyoma virus, so this needs a minimum amount of DNA for extracting and working in PCR.

#### 3- El- Awardy method 2005 (15)

This method designed by El-Awardy and colleagues for extracting DNA from blood, was performed on urine sediment containing defined tachyzoites µl^−1^. This method is very simple especially there is enough DNA for extraction but in the present study because the amount of DNA was very little, it was not a good method.

#### 4- Priem method 1997 (NaOH lysis) (16)

This method is simple and suitable for DNA extraction by using the NaOH solution for cell lyses. It was used by Priem for extraction of Lyme disease agent DNA from urine.

#### 5- Cinnagen^®^ Co. extraction kit

The DNP kit produces by Cinnagen, an Iranian company. It has been designed for extracting DNA from several biological samples and urine as well.

### DNA Amplification

An inventive multiplex semi nested PCR was designed with 3 primers (2 forwards and a common reverse primer) on the basis of a 529 bp fragment of *T. gondii* with 200-300 repetitive frequency (data not shown).

### Data collection

After each extraction in order to assessment and comparing of several extraction methods, using NanoDrop^®^ ND-1000 spectrophotometer, (USB2G32202BE165) determined total DNA in samples and a multiplex semi nested PCR assay. The amount of total DNA in sample elute was assessed by NanoDrop and presence or absence of a specific band in a semi-nested PCR product on agarose gel visualizing with ethydium bromide under UV transiluminator.

## Results

Because having a minimum amount of DNA is essential for all molecular based methods, finding an extraction method with high quality and quantity for DNA extraction of urine was important. Almost extraction methods of urine from literature plus an Iranian company – Cinnagen extraction kit were investigated. The DNA of more than 100 urine samples with known number of tachyzoites was purified comparatively by five better methods. The results reviled that Cinnagen kit performed with more efficacies. It works well up to 1-5 tachyzoites urine. DNA extraction was performed as kit instruction.

The sensitivity, minimum essential tachyzoite number, amount of extracted DNA and PCR results are summarized in Table 1. In addition, to search for finding optimized DNA extraction method, study of some effective factors affected the DNA before and after extraction was done. The PCR inhibitors and DNases are the major factors affecting the DNA-based methods (data not shown).

**Table 1:** Comparative performance and minimum limit of DNA extraction from urine samples by different extraction methods and theirs response to PCR

Extraction method	Tachyzoites/µl 0f urine	Absorbance 260/280 nm	Total extracted DNA(µg)	PCR test	Minimal tachyzoite /µl for a positive PCR result
Fuentes (1996)	10	1.02	2.55	+	5
Holman (2003)	10	1.15	3.67	+	3-5
Awardy(2005)	10	0.88	1.2	−	5-10
Priem (1997)	10	0.65	0.93	−	5-10
Cinnagen kit	10	2	9.9	+	1-5

**Fig. 1:**
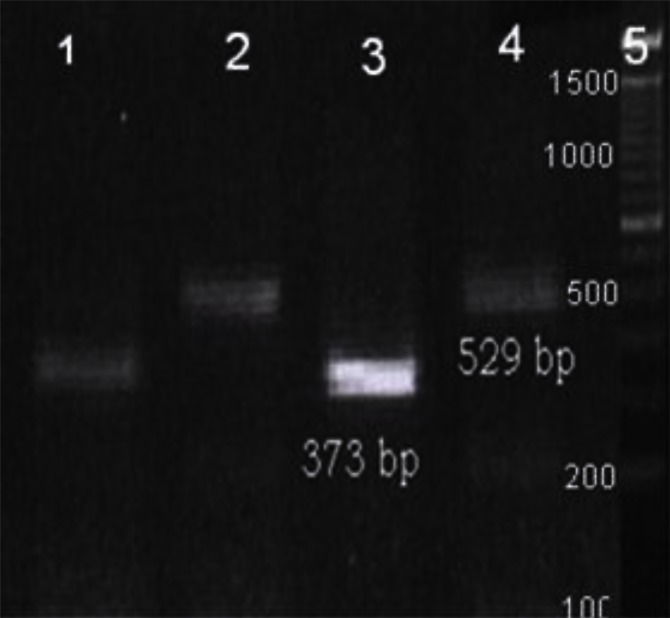
Electrophoresis of PCR product after running on 2% agarose gel with ethydium bromide under UV transilluminator electrophoresis of PCR product of about 2tachyzoite µl^−1^ primary DNA after running on 2% agarose gel with ethydium bromide under UV transillumination. Line 1: current PCR with ToxIF and Tox5 (internal fragment 373 bp), Line 2: current PCR with Tox4 and Tox5 (external fragment 529 bp) Line 3: Multiplex semi nested PCR with Tox 4, Tox IF and Tox5. Line 4: current PCR with ToxIF and Tox5 Line 5: 100 bp ladder DNA size marker. There is a sharp band in the line 3 due to good performance of Multiplex semi nested PCR- a novelty of this study

In order to overcome some unwanted factors, some general rules as taking fresh urine samples and left the samples on ice until PCR, addition of EDTA to urine for chelating the Ca^2 +^ and Mg^2 +^ ions which are critical to work with DNases and avoiding the DNA degradation were used. Moreover a fragment of *T*. *gondii* DNA (AF14765 gene bank) with 250 -300 fold frequency was used to increase the sensitivity of nested PCR up to x10 fold in comparison with other targets like B1 gene. Designing a well worked semi nested PCR was as a novelty of present investigation (Fig. 1)

## Discussion

Among the agents which affect the DNA amount in urine, some have general acceptance and some are controversial (3, 4).

For comparing and finding the optimum urine sampling, collecting, DNA extraction and PCR methods, a critical and accurate measure with good validity and reliability required. Rauter used the threshold number cycle at real time PCR as a measure that is more critical and sensitive than own. He demonstrated that, urine of mid-day is better than the urine of the morning due to accumulation of salt, DNases and some PCR inhibitors in morning (17).

Almost the samples were taken in morning, in the order to setting up the system with the worst urine condition. It would obtain the better results if urine of mid- day were used. Due to some small differences between DNA extraction methods it was difficult to compare all methods absolutely, but there was some priorities with some methods (Table 1). There are many DNA extraction methods in this regard in literature but almost designed for especial purposes and does not work in general (12–17). In the present study, Cinnagen kit worhed with more efficacies among five different extraction methods.

Diagnosis of some infectious diseases with low number or hidden pathogenic agents is critical. The pathogens DNA that released in blood are a few amounts and inter to urine scarcely. So, having a method of purification and extraction DNA with high quality and quantity is an important issue. In present study, toxoplasmosis was used as a model. There is a great deal of experiments on using the urine for diagnosis of different pathogenic agents that cause disseminated infection and their diagnosis depend on invasive samples (2–11).

It is going to perform a molecular based diagnostic PCR method using urine samples from toxoplasmosis suspected persons and serum specific anti- toxoplasma IgM and IgG titer by ELISA comparatively.

Over all, despite of some good critical evaluation of urine for diagnosis of some infectious and non – infectious diseases, it needs more studies for using urine as a routine laboratory sample for molecular-based methods.
